# Impact of continuous glucose monitoring on glycemic control and its derived metrics in type 1 diabetes: a longitudinal study

**DOI:** 10.3389/fendo.2023.1165471

**Published:** 2023-05-15

**Authors:** So Hyun Cho, Seohyun Kim, You-Bin Lee, Sang-Man Jin, Kyu Yeon Hur, Gyuri Kim, Jae Hyeon Kim

**Affiliations:** ^1^ Division of Endocrinology and Metabolism, Department of Medicine, Samsung Medical Center, Sungkyunkwan University School of Medicine, Seoul, Republic of Korea; ^2^ Department of Clinical Research Design and Evaluation, Samsung Advanced Institute for Health Sciences and Technology, Sungkyunkwan University, Seoul, Republic of Korea

**Keywords:** CGM, CGM metrics, T1DM, HbA1c, longitudinal study

## Abstract

**Aim:**

We explored the effectiveness of continuous glucose monitoring for 1 year on glycated A1c reduction in adults with type 1 diabetes mellitus.

**Methods:**

We included type 1 diabetes mellitus adults who were either new continuous glucose monitoring users (*N* = 155) or non-users who were under standard care (*N* = 384). Glycated A1c was measured at baseline and 3, 6, 9, and 12 months. Individuals with (*N* = 155) or without continuous glucose monitoring use (*N* = 310) were matched 1:2 by propensity score. We used the linear mixed models to identify the quantitative reduction in repeated measures of glycated A1c.

**Results:**

The change in glycated A1c from baseline to 12 months was −0.5% ± 1.0% for the continuous glucose monitoring user group (*N* = 155, *P* < 0.001) and −0.01% ± 1.0% for the non-user group (*N* = 310, *P* = 0.816), with a significant difference between the two groups (*P* = 0.003). Changes in glycated A1c were significant at 3, 6, 9, and 12 months compared with those at baseline in patients using continuous glucose monitoring (*P* < 0.001), and the changes differed significantly between the groups (*P* < 0.001). A linear mixed model showed an adjusted treatment group difference in mean reduction in glycated A1c of −0.11% (95% confidence interval, −0.16 to −0.06) each three months. In the continuous glucose monitoring user group, those who achieved more than 70% of time in range significantly increased from 3 months (37.4%) to 12 months (48.2%) (*P* < 0.001).

**Conclusion:**

In this longitudinal study of type 1 diabetes mellitus adults, the use of continuous glucose monitoring for 1 year showed a significant reduction in glycated A1c in real-world practice.

## Introduction

Hemoglobin A1c (HbA1c) has been the gold standard marker for assessing glycemic status and predicting diabetes complications, but it provides limited information that cannot reflect glycemic variability and the presence of severe hypoglycemia or hyperglycemia (1, 2). Continuous glucose monitoring (CGM) demonstrates a continuous measurement of glucose levels over time and detects glucose variations with CGM metrics (3). The main CGM metrics including time in range (TIR), time below range (TBR), and time above range (TAR) inform more personalized glycemic profiles compared with HbA1c (4). Studies have demonstrated that the use of CGM improved glycemic control in patients with type 1 diabetes mellitus (T1DM) using daily insulin injection (5–8). In various diabetes guidelines, the use of CGM is suggested as a standard treatment for the management of T1DM (9, 10). In addition, the use of CGM in T1DM is continuously expanding due to recent improvements in the accuracy and period of use of the sensor and the expansion of the national reimbursement policy (11). However, to the best of our knowledge, longitudinal studies determining the effectiveness of CGM on glycemic control in adults with T1DM under routine clinical care are limited. In the current study, we therefore evaluated the association between CGM use for 1 year and glycemic control among patients with T1DM in a longitudinal real-world setting. In addition, we investigated longitudinal changes of CGM-derived metrics in T1DM patients who used CGM for 1 year.

## Materials and methods

### Study population and study design

**Figure 1:**
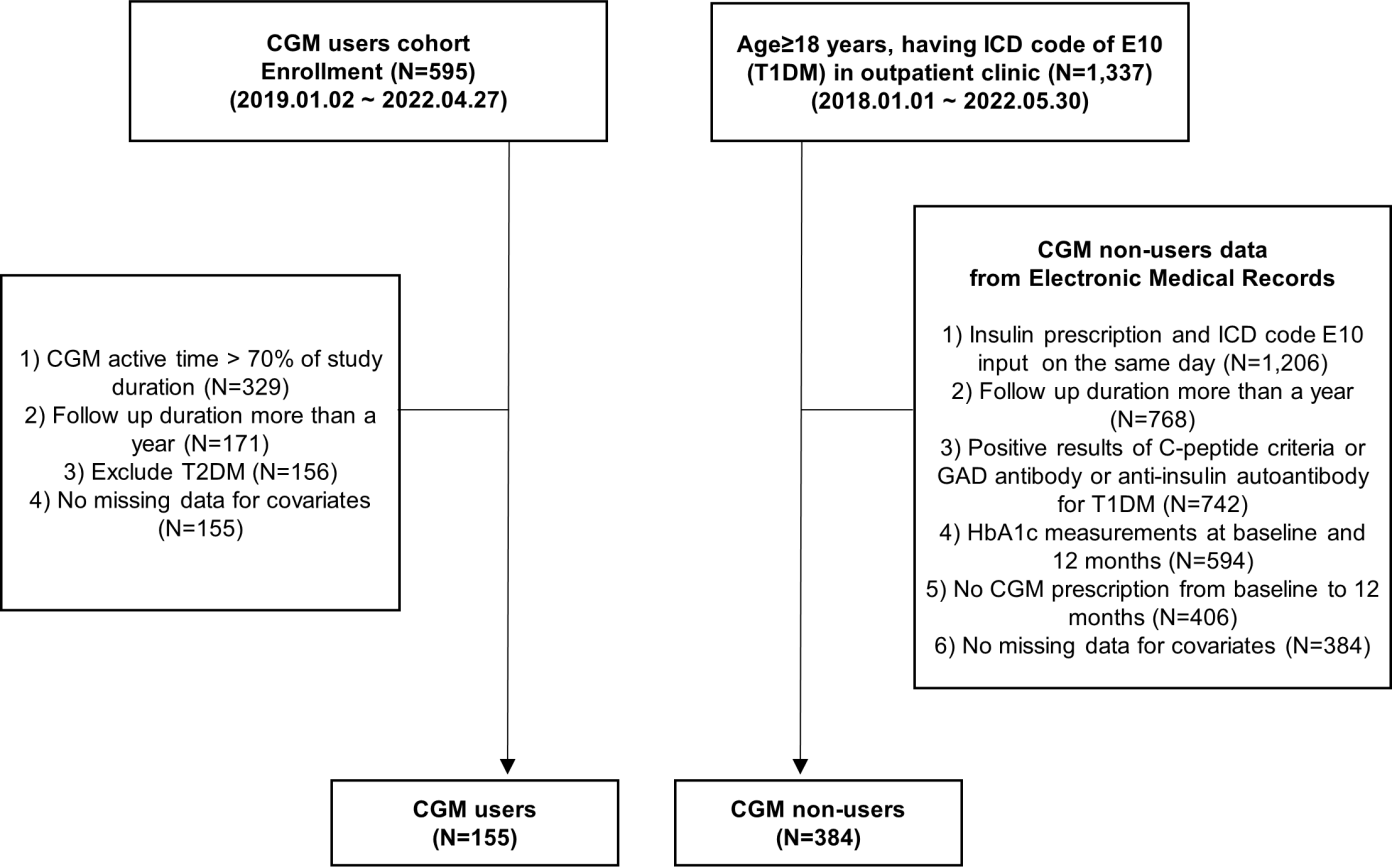
Flowchart. CGM, continuous glucose monitoring; GAD, glutamic acid decarboxylase; HbA1c, hemoglobin A1c; ICD, International Classification of Diseases, T1DM, type 1 diabetes mellitus; T2DM, type 2 diabetes mellitus.

This propensity-matched, single-center, 1-year longitudinal study was conducted at Samsung Medical Center (SMC) in the Republic of Korea. In the CGM user cohort, patients with diabetes mellitus (DM) who visited the outpatient clinic of the Endocrinology Department of SMC between January 2019 and April 2022 and consented to the study requirements to initiate CGM use were prospectively enrolled (*N* = 595, **Figure 1**). The use of CGM was recommended by the physicians and included those who consented to its use. Among them, those who applied CGM for at least 70% of the study period (*N* = 329) and those who continued to visit the outpatient clinic for more than 1 year (*N* = 171) were included. Also, patients with type 2 diabetes mellitus (T2DM) who had ICD-10 (International Classification of Diseases 10th Revision) code of E11–14 (*N* = 15) and missing data for covariates (*N* = 1) were excluded. Finally, a total of 155 people were analyzed as CGM users. They continued to use CGM for 1 year, and HbA1c levels were measured at baseline and 3, 6, 9, and 12 months. CGM data were collected during regular clinical visits each three months for the 12-month study period.

To compare the efficacy of CGM use on glycemic control between CGM users and non-users, data from CGM non-users were also collected retrospectively from the Clinical Data Warehouse (CDW) of SMC electronic medical records (EMR) between January 2018 and May 2022. Among CGM non-users, those who visited the outpatient clinic of the Endocrinology Department and Pediatrics of SMC and those who were 18 years of age or older and who had an E10 ICD code and did not use CGM (*N* = 1,337) were included. Among them, we included T1DM patients who were at least on insulin prescription for T1DM on the same day as E10 ICD code diagnosis; tested positive for GAD II antibody, anti-insulin autoantibody, or C-peptide criteria (fasting C-peptide <0.6 ng/ml or post-stimulated C-peptide <1.8 ng/ml); and had more than a year of outpatient visits with their HbA1c measured at baseline and 12 months (*N* = 594).

We excluded people who had a CGM prescription in the follow-up duration of 1 year (*N* = 188) and missing variables for covariates (*N* = 22). Finally, we analyzed 155 T1DM CGM users and 384 T1DM CGM non-users, respectively (**Figure 1**).

### Clinical and laboratory measurements

The index dates of the CGM user and non-user groups are the date of first use of CGM and the date when insulin and E10 diagnosis were first prescribed on the same day in the study period, respectively. We collected demographic information of the patients, including age, sex, duration of diabetes, total daily dose of insulin use, anthropology measurements of body weight and height, and laboratory values such as HbA1c, estimated glomerular filtration rate (eGFR), alanine aminotransferase (ALT), aspartate aminotransferase (AST), total cholesterol, high-density lipoprotein (HDL) cholesterol, low-density lipoprotein (LDL) cholesterol, and triglyceride (TG) at baseline. Body mass index (BMI) was calculated as body weight (kg) divided by height (m) squared. Blood samples were collected after an overnight fast. HbA1c level as the main outcome of this study was determined by high-performance liquid chromatography on an HLC-723G8 automated glycohemoglobin analyzer (TOSOH, Yokkaichi, Japan). We classified patients with type 1 diabetes by insulin regimen, such as multiple daily injection with rapid-acting analogs and long-acting analogs, mixpen analogs, and continuous subcutaneous insulin infusion.

### CGM parameters

The CGM systems used were real-time Dexcom G5 and G6 (Dexcom, San Diego, CA, USA), Guardian Connect with Enlite Sensor (Medtronic, Northridge, CA, USA), and first-generation FreeStyle Libre System (Abbott Diabetes Care, Witney, UK). We assessed the percentage of time CGM was active during the study period and defined CGM users as those who applied CGM for more than 70% of the study period. The CGM metrics were calculated with percent time in range (TIR) of 70–180 mg/dl, level 2 hyperglycemia of time above range (TAR) >250 mg/dl, level 1 + 2 hyperglycemia of TAR >180 mg/dl, level 1 + 2 hypoglycemia of time below range (TBR) <70 mg/dl, and level 2 hypoglycemia of TBR <54 mg/dl (11). Also, the glucose management indicator (GMI), mean glucose, and glycemic variability such as the coefficient of variation (CV) were assessed (12). The CGM metrics were calculated from the previous 90 days at the 3- and 12-month time points, respectively. In the subgroup analyses of CGM metrics, data from 139 patients were analyzed, excluding individuals who switched type of CGM or changed accounts (*N* = 2) or did not have CGM data for two continuous weeks at 3 and 12 months, respectively (*N* = 14).

### Statistical analysis

Propensity score (PS) matching used to adjust for measured confounding factors was conducted at a 1:2 ratio by using a nearest-neighbor algorithm, and age, sex, and baseline HbA1c were included as covariates. The comparability of covariates between CGM users and non-users was evaluated by standardized mean difference (SMD). An SMD value below 0.1 means the covariates are balanced between groups (13). We calculated the mean and standard deviation of the changes in the HbA1c value of each subsequent observation from the baseline value, and we used a paired *t*-test to determine if the changes were statistically significant. The significance of the difference in the changes in HbA1c levels between the CGM users and non-users was also examined using a two-sample *t*-test.

We used repeated-measures analysis in a linear mixed model (LMM) for longitudinal data, which allows the inclusion of all available data and deals with the missing data. The model showed random intercepts and random slopes with adjustment for confounding factors to identify the quantitative reduction in HbA1c between CGM users and non-users (14). To account for possible baseline confounding factors, we fitted three models with varying degrees of adjustment. Model 1 was a crude model. Model 2 was adjusted for age, sex, baseline HbA1c, and BMI. According to a previous study identifying the significant association between HbA1c and lipid levels, model 3 further was adjusted for the continuous value of eGFR, total cholesterol, HDL cholesterol, LDL cholesterol, TG, duration of diabetes, total daily dose of insulin use, and insulin regimen (15). Multiple linear regression analysis was conducted to identify the predictive marker for a better reduction in HbA1c from baseline to 12 months in baseline variables among individuals with CGM use (*N* = 155). In the sensitivity analysis, we retrospectively included all CGM users with T1DM during the same study period. We collected all variables mentioned above, as well as follow-up HbA1c values at baseline and 3, 6, 9, and 12 months. We also fitted the adjusted LMM to explore the association between the use of CGM and HbA1c. In the analysis for metrics of CGM, continuous variables were presented as mean ± SD or numbers (%), and categorical variables were expressed as ratios of percentages. Continuous variables were compared using paired *t*-test and *χ* ^2^ test between 3 and 12 months. Comparisons of CGM metrics measured from 3 to 12 months were calculated using the linear mixed-effect analysis for continuous variables and the Cochran–Armitage trend test for categorical variables. *P*-values <0.05 were considered statistically significant, and analyses were performed using SPSS software ver. 27.0 (IBM, Armonk, NY, USA) and R Studio version 1.4.1103 software.

## Results

### Baseline characteristics of the study population

The baseline characteristics of the study population are presented in **Table 1**. Before PS matching, 155 and 384 patients were classified as CGM users and non-users, respectively. After 1:2 PS matching, 155 CGM users and 310 CGM non-users were finally analyzed. The SMD of matching variables including age, sex, and baseline HbA1c was below 0.1 and comparable between the two groups. The mean age of the study population was 43.4 ± 15.7 years, and 46% were men. The mean duration of diabetes and the total daily dose of insulin of the participants were 12.8 ± 9.3 years and 43.6 ± 24.2 units, respectively. The mean baseline BMI was 23 ± 3.5 kg/m^2^, and the mean baseline HbA1c level was 7.5% ± 1.2% in both groups.

**Table 1:** Baseline characteristics of the study participants after propensity score matching (*n* = 465).

	CGM non-users (*n* = 310)	CGM users (*n* = 155)	*P*-value
Age, years	43.4 ± 16.6	43.5 ± 13.6	0.954
Female, %	165 (53.2%)	86 (55.5%)	0.717
Duration of diabetes, years	13.7 ± 9.5	10.9 ± 8.6	0.002
Total daily dose of insulin, units	45.2 ± 25.1	40.4 ± 22.0	0.042
Hemoglobin A1c, %	7.5 ± 1.2	7.5 ± 1.2	0.85
BMI, kg/m^2^	23.2 ± 3.8	22.7 ± 3.1	0.143
eGFR, ml/min/1.73 m^2^	94.0 ± 28.3	98.1 ± 24.4	0.112
ALT, U/L	21.3 ± 14.7	21.3 ± 16.8	0.988
AST, U/L	24.1 ± 14.8	24.4 ± 21.5	0.892
Total cholesterol, mg/dl	158.9 ± 35.4	172.3 ± 88.1	0.071
HDL cholesterol, mg/dl	64.0 ± 18.7	68.8 ± 17.4	0.007
LDL cholesterol, mg/dl	94.3 ± 34.5	95.7 ± 33.1	0.685
TG, mg/dl	95.6 ± 60.3	84.3 ± 45.1	0.023
Insulin regimen (%)			0.064
Multiple daily injections	283 (91.3%)	149 (96.1%)	
Mixpen analogs	9 (2.9%)	0 (0%)	
Continuous subcutaneous insulin infusion	18 (5.8%)	6 (3.9%)	
Type of CGM (%)			–
Abbott Freestyle Libre	–	25 (16.1%)	
Dexcom G5	–	100 (64.5%)	
Dexcom G6	–	15 (9.7%)	
Medtronic Guardian Connect	–	15 (9.7%)	

Data are expressed as mean ± standard deviation (SD) or n (%).

ALT, alanine aminotransferase; AST, aspartate aminotransferase; BMI, body mass index; CGM, continuous glucose monitoring; eGFR, estimated glomerular filtration rate; HDL, high-density lipoprotein; LDL, low-density lipoprotein; SD, standard deviation; TG, triglyceride.

### Changes in HbA1c

**Figure 2:**
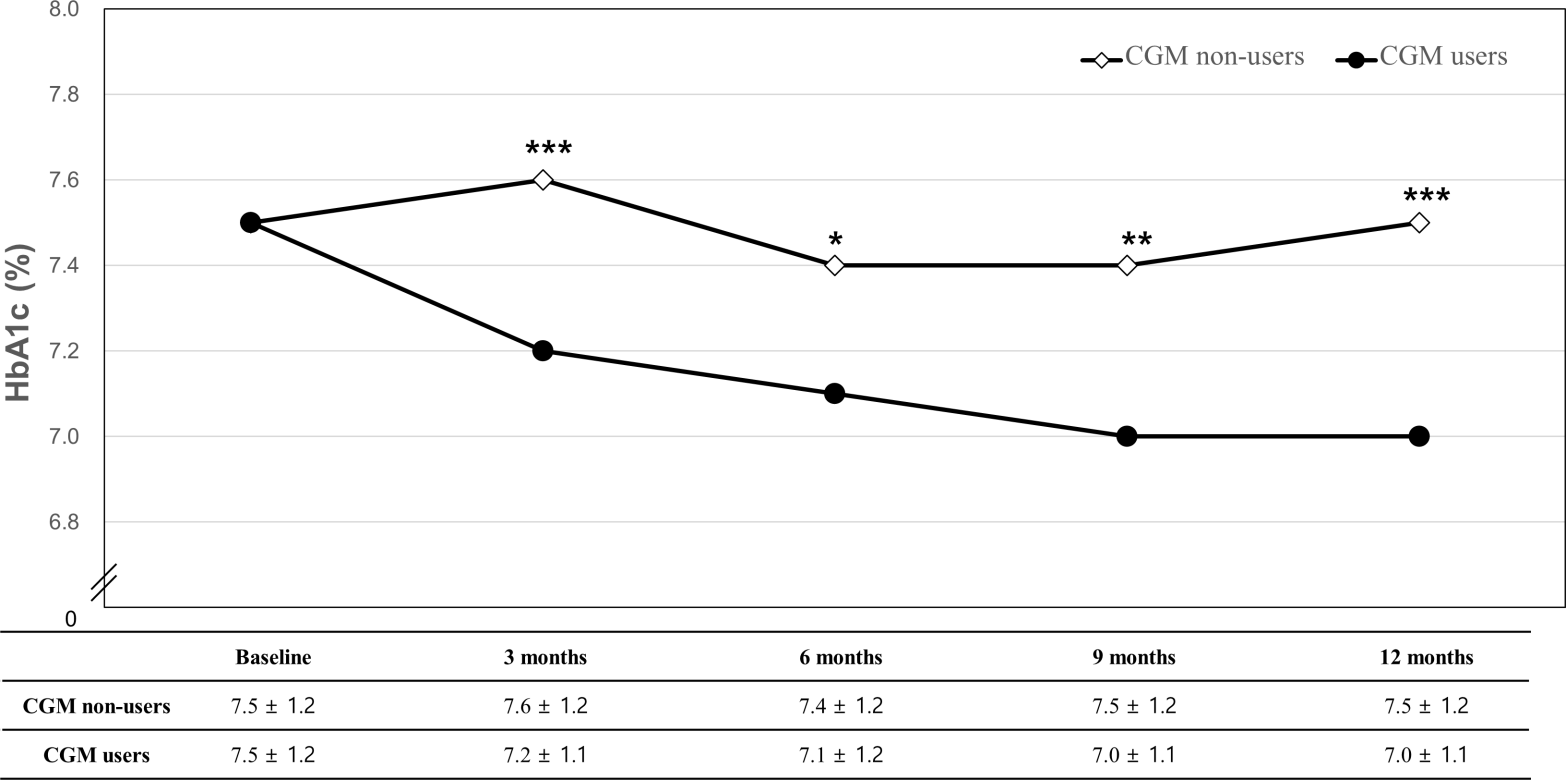
Changes in HbA1c from baseline to 12 months. *P-value for the mean difference between CGM non-users and users (*P < 0.05, **P < 0.01, ***P < 0.001).

Changes in HbA1c from baseline to each follow-up in CGM users and non-users are described in **Figure 2** and **Table 2**. The changes in HbA1c from baseline to 12 months were −0.5% ± 1.0% for the CGM user group (*P* < 0.001) and −0.01% ± 1.0% for the CGM non-user group (*P* = 0.816) with significant difference between the two groups (*P* = 0.003). The levels of HbA1c were significantly decreased at 3 months compared with baseline by −0.4% ± 0.8% in the CGM user group (*P* < 0.001) with significant between-group differences (*P* < 0.001). In all follow-up study periods, CGM users showed significant improvement in HbA1c levels from baseline and the changes differed significantly between the two groups.

**Table 2:** Changes in HbA1c at each follow-up.

	CGM non-users		CGM users		*P*-value
	*N*	HbA1c (%)	*N*	HbA1c (%)	
Baseline (mean ± SD)	310	7.5 ± 1.2	155	7.5 ± 1.2	
At 3 months (mean ± SD)	256	7.6 ± 1.2	155	7.2 ± 1.1	
Changes from baseline to 3 months (mean ± SD)	−0.1 ± 1.0		−0.4 ± 0.8		<0.001^b^
* P*-value for the mean difference from baseline to 3 months	0.324^a^		<0.001^a^		
At 6 months (mean ± SD)	230	7.4 ± 1.2	154	7.1 ± 1.2	
Changes from baseline to 6 months (mean ± SD)	−0.1 ± 1.0		−0.4 ± 1.0		0.003^b^
* P*-value for the mean difference from baseline to 6 months	0.09^a^		<0.001^a^		
At 9 months (mean ± SD)	210	7.5 ± 1.2	155	7.0 ± 1.1	
Changes from baseline to 9 months (mean ± SD)	−0.2 ± 1.1		−0.5 ± 1.0		0.007^b^
* P*-value for the mean difference from baseline to 9 months	0.019^a^		<0.001^a^		
At 12 months (mean ± SD)	310	7.5 ± 1.2	155	7.0 ± 1.1	
Changes from baseline to 12 months (mean ± SD)	−0.01 ± 1.0		−0.5 ± 1.0		0.003^b^
* P*-value for the mean difference from baseline to 12 months	0.816^a^		<0.001^a^		

CGM, continuous glucose monitoring; HbA1c, hemoglobin A1c; SD, standard deviation.

^a^P-values were derived from a paired t-test.

^b^P-values were derived from a two-sample t-test.

### The average difference in decreasing HbA1c each three months in patients with and without CGM at baseline

The LMM showed that the overall average values in decreasing HbA1c each three months in patients with and without CGM were −0.01% [95% confidence interval (95% CI), −0.04 to 0.02; *P* = 0.61] and −0.12% (95% CI, −0.16 to −0.08; *P* < 0.001), respectively (**Table 3**). The treatment group difference in mean reduction in HbA1c each three months was −0.11% (95% CI, −0.16 to −0.06; *P* < 0.001) after adjustment for age, sex, baseline HbA1c, BMI, eGFR, total cholesterol, HDL cholesterol, LDL cholesterol, TG, and insulin regimen (model 3).

**Table 3:** Results of the linear mixed model of the effect of CGM on HbA1c change.

	CGM non-users (*n* = 310) (*β*, 95% CI)	CGM users (*n* = 155) (*β*, 95% CI)	*P*-value
**Overall average reduction of HbA1c (%) each three months**	−0.01 (−0.04, 0.02)	−0.12 (−0.16, −0.08)	<0.001
Average difference of HbA1c (%) each three months			
**Model 1**	0 (reference)	−0.11 (−0.16, −0.07)	<0.001
**Model 2**	0 (reference)	−0.11 (−0.16, −0.06)	<0.001
**Model 3**	0 (reference)	−0.11 (−0.16, −0.06)	<0.001

Model 1 is the unadjusted model; model 2 is adjusted for age, sex, BMI, and baseline HbA1c; model 3 is further adjusted for eGFR, total cholesterol, HDL, LDL, TG, duration of diabetes, total daily dose of insulin, and insulin regimen.

BMI, body mass index; CGM, continuous glucose monitoring; CI, confidence interval; eGFR, estimated glomerular filtration rate; HbA1c, hemoglobin A1c; HDL, high-density lipoprotein; LDL, low-density lipoprotein; TG, triglyceride.

### Sensitivity analysis

In the sensitivity analysis, the overall average values each three months of reduction in HbA1c in patients with and without CGM were −0.02% (95% CI, −0.05 to 0.004) and −0.12% (95% CI, −0.14 to −0.09), respectively (**Supplementary Table 1**). The adjusted treatment group difference in mean reduction in HbA1c each three months in model 3 was −0.09% (95% CI, −0.13 to −0.05).

### Analysis of baseline characteristics associated with better HbA1c reduction by CGM use

To identify the predictive marker of CGM use on HbA1c reduction from baseline to 12 months, multiple linear regression was conducted in individuals with CGM use (**Supplementary Table 2**). After adjustment for age, sex, BMI, eGFR, total cholesterol, HDL, LDL, TG, duration of diabetes, total daily dose of insulin, and insulin regimen, higher baseline HbA1c was a predictive marker for a better HbA1c reduction glycemic control following CGM use [*β* (95% CI) = −0.433 (−0.545, −0.321), *P* < 0.001].

### Analysis of CGM-derived metrics

In the analysis of CGM-derived metrics, CGM users (*N* = 139) having ambulatory glucose profile (AGP) data at 3 and 12 months were included. **Table 4** shows the difference in CGM-derived metrics at 3 and 12 months and the proportions of participants who achieved the CGM metrics goals (TIR > 70%, TBR < 70 mg/dl < 4%, TBR < 54 mg/dl < 1%, TAR > 180 mg/dl < 25%, TAR > 250 mg/dl < 5%, CV ≤ 36%, GMI ≤ 7%) in accordance with the international consensus recommendations.

**Table 4:** Changes of CGM-derived metrics in the CGM user group at baseline and the endpoint.

Variables	3 months	6 months	9 months	12 months	*P*-value^a^	*P*-value^b^
	(*N* = 139)	(*N* = 128)	(*N* = 128)	(*N* = 139)		
**TIR (%)**	64.5 ± 17.7	64.0 ± 19.2	66.9 ± 17.5	67.4 ± 18.2	0.002	0.001
**TIR >70%**	37.4% (*N* = 52)	39.1% (*N* = 50)	44.5% (*N* = 57)	48.2% (*N* = 67)	<0.001	0.045
**TBR (<54 mg/dl) (%)**	0.8 ± 2.1	1.2 ± 4.0	0.9 ± 1.6	0.7 ± 1.5	0.293	0.328
**TBR-54 <1%**	77.0% (*N* = 107)	74.2% (*N* = 95)	73.4% (*N* = 94)	82.0% (*N* = 114)	<0.001	0.367
**TBR (<70 mg/dl) (%)**	3.4 ± 4.5	4.1 ± 6.2	3.6 ± 4.3	3.0 ± 3.3	0.198	0.214
**TBR-70 <4%**	74.8% (*N* = 104)	68.0% (*N* = 87)	70.3% (*N* = 90)	68.4% (*N* = 95)	<0.001	0.317
**TAR (>180 mg/dl) (%)**	31.9 ± 18.7	31.9 ± 20.7	29.3 ± 18.6	29.5 ± 18.7	0.021	0.010
**TAR-180 <25%**	36.0% (*N* = 50)	40.6% (*N* = 52)	46.1% (*N* = 59)	43.9% (*N* = 61)	<0.001	0.122
**TAR (>250 mg/dl) (%)**	10.9 ± 11.0	11.2 ± 12.8	9.3 ± 10.0	9.5 ± 11.9	0.032	0.012
**TAR-250 <5%**	35.3% (*N* = 49)	38.3% (*N* = 49)	49.2% (*N* = 63)	47.5% (*N* = 66)	<0.001	0.012
**CV (%)**	35.8 ± 5.9	35.7 ± 6.1	35.5 ± 5.8	34.9 ± 6.1	0.014	0.023
**CV ≤36%**	50.4% (*N* = 70)	49.2% (*N* = 63)	53.1% (*N* = 68)	58.3% (*N* = 81)	<0.001	0.147
**GMI (%)**	7.2 ± 1.0	7.2 ± 1.1	7.1 ± 1.0	7.1 ± 1.1	0.078	0.046
**GMI ≤7%**	46.8% (*N* = 65)	50.8% (*N* = 65)	53.9% (*N* = 69)	55.4% (*N* = 77)	<0.001	0.128
**Mean glucose (mg/dl)**	160.2 ± 32.8	159.5 ± 37.6	155.7 ± 31.4	157.1 ± 32.8	0.073	0.043
**Active percentage of CGM (%)**	84.1 ± 16.2	84.1 ± 16.9	87.5 ± 14.0	87.5 ± 14.2	0.046	0.020
**Median days**	74.9 ± 15.6	75.7 ± 15.2	76.9 ± 15.8	77.8 ± 14.4	0.088	0.080

CGM, continuous glucose monitoring; CV, coefficient of variation; GMI, glucose management indicator; TAR, time above range; TBR, time below range; TIR, time in range.

^a^Paired t-test for continuous variables and χ^2^ test for categorical variables between 3 and 12 months.

^b^Linear mixed-effect analysis for continuous variables and the Cochran–Armitage trend test for categorical variables from 3 to 12 months.

The variables of TIR, TAR >180 mg/dl, TAR >250 mg/dl, and CV at 12 months showed significant improvements compared with the first 3 months (*P* = 0.002, *P* = 0.021, *P* = 0.032, and *P* = 0.014, respectively). The proportions of individuals with TIR >70% at 3 and 12 months were 37.4% and 48.2%, respectively (*P* < 0.001). The proportions of individuals who achieved target goals of TBR <54 mg/dl, TAR >180 mg/dl, TAR >250 mg/dl, CV, and GMI values at 12 months were significantly increased compared with the first 3 months (*P* < 0.001).

## Discussion

In this real-world propensity-matched cohort comparison study with T1DM patients under routine clinical practice, CGM use over 1 year showed a significant reduction in HbA1c level of 0.5% from baseline compared with PS-matched CGM non-users with a significant difference between the two groups (*P* = 0.003). In addition, a longitudinal LMM showed that the main effect of CGM on HbA1c change was significant in CGM users compared with non-users after adjusting for multivariable confounders. Furthermore, in the analysis of CGM-derived metrics in the CGM user group, we demonstrated a significant improvement in TIR (*P* = 0.002) in the last 3 at 12 months compared with the first 3 months, and the proportion of those who achieved target goals of TIR >70%, TBR <54 mg/dl <1%, TAR >180 mg/dl <25%, TAR >250 mg/dl <5%, CV ≤36%, and GMI ≤7% was higher over the last 3 and 12 months than the first 3 months (all *P* < 0.001).

These results are in line with previous studies (5, 16–18). A previous randomized clinical trial (RCT) from the United States reported that the use of CGM (*N* = 105) compared with usual care (*N* = 53) resulted in a greater decrease in HbA1c levels over the course of 24 weeks in T1DM patients who required multiple daily insulin injections (5). The adjusted treatment group difference in mean change in HbA1c level from baseline was −0.6% (95% CI, −0.8% to −0.3%; *P* < 0.001). Also, one meta-analysis of RCTs comparing CGM to self-monitoring of blood glucose (SMBG) in patients with T1DM reported a significantly lower HbA1c at the endpoint with a duration of at least 12 weeks (−0.2%, 95% CI, −0.3% to −0.1%) (17). Recently, one retrospective study reported a statistical decrease in HbA1c using real-time CGM compared with non-initiators (19). This trial of real-time CGM initiators (*N* = 3,806) *vs*. non-initiators (*N* = 37,947) who received care in Northern California (2014–2019) for insulin-treated diabetes reported a decrease in mean HbA1c from 8.2% to 7.8% in the CGM group and 8.3% to 8.2% in the control group over 12 months (difference-in-differences estimate, −0.4%; 95% CI, −0.5% to −0.3%; *P* < 0.001). Also, hypoglycemia rates increased among non-initiators from 1.9% to 2.3% while falling in CGM initiators from 5.1% to 3.0% (difference-in-differences estimate, −2.7%; 95% CI, −4.4% to −1.1%; *P* = 0.001). In our study, we prospectively enrolled Asians, particularly Korean T1DM patients who were CGM users, and used strict inclusion criteria of T1DM using not only diagnostic codes but also C-peptide levels, autoantibodies, and consistent insulin use. We further conducted a LMM analysis to show that the effect of CGM use on HbA1c change was independently significant in CGM users compared with non-users after adjusting for multivariable confounders.

In terms of response to treatment, HbA1c changes were evaluated, and a greater reduction in HbA1c in patients with higher HbA1c at baseline has been revealed in a previous meta-analysis (20). The weighted *R* ^2^ value assessing the association between baseline HbA1c and absolute change in HbA1c was 0.485 (*P* < 0.001). As the American Diabetes Association recommends a general goal for glycemic control of HbA1c <7% for T1DM patients, it is especially notable that the HbA1c level at baseline was 7.5% in our study, which is closer to the target level. Moreover, we also found that among CGM users, the proportions of individuals with TIR >70% at 3 and 12 months were 37.4% (52 of 139) and 48.2% (67 of 139), respectively (*P* < 0.001). The correlation between TIR and chronic complications in T1DM was suggested by several studies including the Diabetes Control and Complications Trial (DCCT), as well as between TIR and HbA1c (21–23). One study using the DCCT dataset evaluated the association of TIR from the seven-point glycemic profiles with the development of DM complications (24). For every 10% lowering of TIR, the hazard rate of development of retinopathy progression was increased by 64% (95% CI, 51% to 78%), and the rate of microalbuminuria development was increased by 40% (95% CI, 25% to 56%). Yapanis and colleagues analyzed 34 publications, mostly cross-sectional studies, including a total of 20,852 participants, and presented the association between CGM-derived measures and diabetes-related complications (25). Higher TIR was related to a low risk of retinopathy, albuminuria, and abnormal carotid intima–media thickness. Also, glycemic variability such as standard deviation of blood glucose levels (SD) and mean amplitude of glycemic excursions (MAGE) was associated with peripheral neuropathy. Our study found that the CV at 12 months (34.9% ± 6.1%) showed a significant decrease compared with that at the first 3 months (35.8% ± 5.9%) (*P* = 0.014). These data also support that glycemic variability is lowered when using a personal CGM in a longitudinal real-world setting (26, 27). In this line, using a personal CGM is associated with a better HbA1c level and CGM-derived metrics of glycemic variability, and further longitudinal studies are required to confirm its relationship with diabetes-related complications in patients with T1DM.

In previous randomized trials of adults with T1DM, CGM use reduced the number of hypoglycemic events (28–30). The SILVER study supported the beneficial long-term effects of CGM on HbA1c and hypoglycemia in people with T1DM. In this RCT, 107 patients with T1DM continued to use CGM over 1 year, and the HbA1c showed a decrease of 0.4% (95% CI, 0.2% to 0.5%). The time spent in hypoglycemia (<54 and <72 mg/dl) decreased from 2.1% to 0.6% (*P* < 0.001) and from 5.4% to 2.9% (*P* < 0.001), respectively (31). Because lowering HbA1c may be associated with an increased risk of hypoglycemia in T1DM, it is important to lower the average blood glucose levels while minimizing hypoglycemia. This was demonstrated in our real-world study by improvements in HbA1c, TIR, and TBR when using CGM over 1 year. Although not all CGM-derived metrics improved statistically, after the use of CGM for 1 year, there was a significant increase in those who reached target goals of the CGM core metrics such as TIR, TBR, TAR, CV, and GMI according to the 2019 consensus statement (32).

The strength of this study is its prospective analysis of CGM use in an observational study in a real-world setting to determine if CGM use promotes the reduction of HbA1c level in T1DM patients. Also, with follow-up of CGM users, we continuously collected CGM-derived metrics data over 1 year in a clinical setting. Also, a LMM was used to consider missing data and examine the trend after adjustment for multivariable confounders. Furthermore, sensitivity analysis was performed to identify the robustness of our findings for the longitudinal effectiveness of CGM use on lowering HbA1c in comparison with those prospectively enrolled. This analysis included all T1DM patients who used CGM based on a retrospective review of EMR. We also further investigated a predictive marker of CGM use on HbA1c reduction from baseline to 12 months in patients with CGM use and found that patients with higher baseline HbA1c had a greater HbA1c reduction after 12 months of CGM use.

However, there are some limitations to this study. First, this was based on Korean individuals at a single center, which may limit the ability to generalize our results to other settings. Second, CGM users may be a group of motivated individuals and that may cause a selection bias, and applying CGM itself may have affected the behavioral change of the patients and lifestyle modification.

Also, CGM users were enrolled prospectively and may have influenced the results, whereas the non-CGM users were reviewed retrospectively. Third, the different types of CGM may have different results in the CGM metrics. Further long-term follow-up trials on the use of CGM in patients with T1DM and analyses of real-world data are needed.

## Conclusions

In conclusion, our longitudinal study of real-world data from T1DM patients over 12 months demonstrated significant improvements in HbA1c among CGM users compared with non-users.

## Data availability statement

The original contributions presented in the study are included in the article/**Supplementary Materials**, further inquiries can be directed to the corresponding authors.

## Ethics statement

The studies involving human participants were reviewed and approved by the Institutional Review Board (IRB) of Samsung Medical Center (approval no. SMC 2018-17-132, 2022-07-110). All patients who were classified as CGM users provided informed, signed consent to participate in this study. An informed consent exemption for non-CGM users was granted by the IRB because all data provided by the CDW of SMC to researchers were de-identified. Written informed consent for participation was not required for this study in accordance with the national legislation and the institutional requirements.

## Author contributions

JK conceived this study. GK and JK contributed to the design of the study. SC and SK conducted the data collection and analysis. SC and SK interpreted the results and wrote the main manuscript text. Y-BL, S-MJ, KH, GK and JK edited the manuscript and contributed to the discussion. All authors contributed to the article and approved the submitted version.

## Supplementary material

Supplementary Figure 1 Flow chart of sensitivity analysis. CGM, continuous glucose monitoring; GAD, glutamic acid decarboxylase; HbA1c, glycated hemoglobin; ICD, International Classification of Diseases; T1DM, Type 1 diabetes mellitus; T2DM, Type 2 diabetes mellitus.

## References

[1] Wright LA Hirsch IB. Metrics beyond hemoglobin A1C in diabetes management: time in range, hypoglycemia, and other parameters. Diabetes Technol Ther (2017) 19(S2):S16–s26. doi: 10.1089/dia.2017.0029 28541136 PMC5444503

[2] Kilpatrick ES Rigby AS Goode K Atkin SL. Relating mean blood glucose and glucose variability to the risk of multiple episodes of hypoglycaemia in type 1 diabetes. Diabetologia (2007) 50(12):2553–61. doi: 10.1007/s00125-007-0820-z 17882397

[3] Nathan DM Cleary PA Backlund JY Genuth SM Lachin JM Orchard TJ. Intensive diabetes treatment and cardiovascular disease in patients with type 1 diabetes. New Engl J Med (2005) 353(25):2643–53. doi: 10.1056/NEJMoa052187 PMC2637991 16371630

[4] Chehregosha H Khamseh ME Malek M Hosseinpanah F Ismail-Beigi F. A view beyond HbA1c: role of continuous glucose monitoring. diabetes therapy : research, treatment and education of diabetes and related disorders. Diabetes ther (2019) 10(3):853–63. doi: 10.1007/s13300-019-0619-1 PMC6531520 31037553

[5] Beck RW Riddlesworth T Ruedy K Ahmann A Bergenstal R Haller S. Effect of continuous glucose monitoring on glycemic control in adults with type 1 diabetes using insulin injections: the DIAMOND randomized clinical trial. Jama (2017) 317(4):371–8. doi: 10.1001/jama.2016.19975 28118453

[6] Bolinder J Antuna R Geelhoed-Duijvestijn P Kröger J Weitgasser R. Novel glucose-sensing technology and hypoglycaemia in type 1 diabetes: a multicentre, non-masked, randomised controlled trial. Lancet (London England) (2016) 388(10057):2254–63. doi: 10.1016/S0140-6736(16)31535-5 27634581

[7] van Beers CA DeVries JH Kleijer SJ Smits MM Geelhoed-Duijvestijn PH Kramer MH. Continuous glucose monitoring for patients with type 1 diabetes and impaired awareness of hypoglycaemia (IN CONTROL): a randomised, open-label, crossover trial. Lancet Diabetes Endocrinol (2016) 4(11):893–902. doi: 10.1016/S2213-8587(16)30193-0 27641781

[8] Weiss R Garg SK Bode BW Bailey TS Ahmann AJ Schultz KA. Hypoglycemia reduction and changes in hemoglobin A1c in the ASPIRE in-home study. Diabetes Technol Ther (2015) 17(8):542–7. doi: 10.1089/dia.2014.0306 PMC4528987 26237308

[9] Holt RIG DeVries JH Hess-Fischl A Hirsch IB Kirkman MS Klupa T. The management of type 1 diabetes in adults. a consensus report by the American diabetes association (ADA) and the European association for the study of diabetes (EASD). Diabetes Care (2021) 44(11):2589–625. doi: 10.1007/s00125-021-05568-3 34593612

[10] Hur KY Moon MK Park JS Kim SK Lee SH Yun JS. Clinical practice guidelines for diabetes mellitus of the Korean diabetes association. Diabetes Metab J (2021) 45(4):461–81. doi: 10.4093/dmj.2021.0156 PMC8369224 34352984

[11] Yoo JH Kim JH. Time in range from continuous glucose monitoring: a novel metric for glycemic control. Diabetes Metab J (2020) 44(6):828–39. doi: 10.4093/dmj.2020.0257 PMC7801761 33389957

[12] Draznin B Aroda VR Bakris G Benson G Brown FM Freeman R. 6. glycemic targets: standards of medical care in diabetes-2022. Diabetes Care (2022) 45(Suppl 1):S83–s96. doi: 10.2337/dc22-S006 34964868

[13] Austin PC. Balance diagnostics for comparing the distribution of baseline covariates between treatment groups in propensity-score matched samples. Stat Med (2009) 28(25):3083–107. doi: 10.1002/sim.3697 PMC3472075 19757444

[14] Sinn DH Kang D Chang Y Ryu S Gu S Kim H. Non-alcoholic fatty liver disease and progression of coronary artery calcium score: a retrospective cohort study. Gut (2017) 66(2):323–9. doi: 10.1136/gutjnl-2016-311854 27599521

[15] Borg R Kuenen JC Carstensen B Zheng H Nathan DM Heine RJ. HbA_1_ (c) and mean blood glucose show stronger associations with cardiovascular disease risk factors than do postprandial glycaemia or glucose variability in persons with diabetes: the A1C-derived average glucose (ADAG) study. Diabetologia (2011) 54(1):69–72. doi: 10.1007/s00125-010-1918-2 20886203 PMC2995856

[16] Haviland N Walsh J Roberts R Bailey TS. Update on clinical utility of continuous glucose monitoring in type 1 diabetes. Curr Diabetes Rep (2016) 16(11):115. doi: 10.1007/s11892-016-0808-5 27718171

[17] Dicembrini I Cosentino C Monami M Mannucci E Pala L. Effects of real-time continuous glucose monitoring in type 1 diabetes: a meta-analysis of randomized controlled trials. Acta diabetologica (2021) 58(4):401–10. doi: 10.1007/s00592-020-01589-3 32789691

[18] Maiorino MI Signoriello S Maio A Chiodini P Bellastella G Scappaticcio L. Effects of continuous glucose monitoring on metrics of glycemic control in diabetes: a systematic review with meta-analysis of randomized controlled trials. Diabetes Care (2020) 43(5):1146–56. doi: 10.2337/dc19-1459 32312858

[19] Karter AJ Parker MM Moffet HH Gilliam LK Dlott R. Association of real-time continuous glucose monitoring with glycemic control and acute metabolic events among patients with insulin-treated diabetes. Jama (2021) 325(22):2273–84. doi: 10.1001/jama.2021.6530 PMC8173463 34077502

[20] Giugliano D Maiorino M Bellastella G Chiodini P Esposito K. Relationship of baseline HbA1c, HbA1c change and HbA1c target of < 7% with insulin analogues in type 2 diabetes: a meta-analysis of randomised controlled trials. Int J Clin Pract (2011) 65(5):602–12. doi: 10.1111/j.1742-1241.2010.02619.x 21489084

[21] Beck RW Bergenstal RM Cheng P Kollman C Carlson AL Johnson ML. The relationships between time in range, hyperglycemia metrics, and HbA1c. J Diabetes Sci Technol (2019) 13(4):614–26. doi: 10.1177/1932296818822496 PMC6610606 30636519

[22] Hirsch IB Welsh JB Calhoun P Puhr S Walker TC Price DA. Associations between HbA(1c) and continuous glucose monitoring-derived glycaemic variables. Diabetic Med (2019) 36(12):1637–42. doi: 10.1111/dme.14065 PMC6899839 31267573

[23] Vigersky RA McMahon C. The relationship of hemoglobin A1C to time-in-Range in patients with diabetes. Diabetes Technol Ther (2019) 21(2):81–5. doi: 10.1089/dia.2018.0310 30575414

[24] Beck RW Bergenstal RM Riddlesworth TD Kollman C Li Z Brown AS. Validation of time in range as an outcome measure for diabetes clinical trials. Diabetes Care (2019) 42(3):400–5. doi: 10.2337/dc18-1444 PMC6905478 30352896

[25] Yapanis M James S Craig ME O'Neal D Ekinci EI. Complications of diabetes and metrics of glycemic management derived from continuous glucose monitoring. J Clin Endocrinol Metab (2022) 107(6):e2221–e36. doi: 10.1210/clinem/dgac034 PMC9113815 35094087

[26] Lind M Polonsky W Hirsch IB Heise T Bolinder J Dahlqvist S. Continuous glucose monitoring vs conventional therapy for glycemic control in adults with type 1 diabetes treated with multiple daily insulin injections: the GOLD randomized clinical trial. Jama (2017) 317(4):379–87. doi: 10.1001/jama.2016.19976 28118454

[27] El-Laboudi AH Godsland IF Johnston DG Oliver NS. Measures of glycemic variability in type 1 diabetes and the effect of real-time continuous glucose monitoring. Diabetes Technol Ther (2016) 18(12):806–12. doi: 10.1089/dia.2016.0146 27996321

[28] Heinemann L Freckmann G Ehrmann D Faber-Heinemann G Guerra S Waldenmaier D. Real-time continuous glucose monitoring in adults with type 1 diabetes and impaired hypoglycaemia awareness or severe hypoglycaemia treated with multiple daily insulin injections (HypoDE): a multicentre, randomised controlled trial. Lancet (London England) (2018) 391(10128):1367–77. doi: 10.1016/S0140-6736(18)30297-6 29459019

[29] Beck RW Hirsch IB Laffel L Tamborlane WV Bode BW Buckingham B. The effect of continuous glucose monitoring in well-controlled type 1 diabetes. Diabetes Care (2009) 32(8):1378–83. doi: 10.2337/dc09-0108 PMC2713649 19429875

[30] Battelino T Phillip M Bratina N Nimri R Oskarsson P Bolinder J. Effect of continuous glucose monitoring on hypoglycemia in type 1 diabetes. Diabetes Care (2011) 34(4):795–800. doi: 10.2337/dc10-1989 21335621 PMC3064030

[31] Lind M Ólafsdóttir AF Hirsch IB Bolinder J Dahlqvist S Pivodic A. Sustained intensive treatment and long-term effects on HbA(1c) reduction (SILVER study) by CGM in people with type 1 diabetes treated with MDI. Diabetes Care (2021) 44(1):141–9. doi: 10.2337/dc20-1468 33199470

[32] Battelino T Danne T Bergenstal RM Amiel SA Beck R Biester T. Clinical targets for continuous glucose monitoring data interpretation: recommendations from the international consensus on time in range. Diabetes Care (2019) 42(8):1593–603. doi: 10.2337/dci19-0028 PMC6973648 31177185

