# Supplementary figures and images for: Impact of continuous glucose monitoring on glycemic control and its derived metrics in type 1 diabetes: a longitudinal study
Source: Front Endocrinol (Lausanne). 2023 May 15;14:1165471. doi: 10.3389/fendo.2023.1165471 (PMC10225713; doi:10.3389/fendo.2023.1165471)

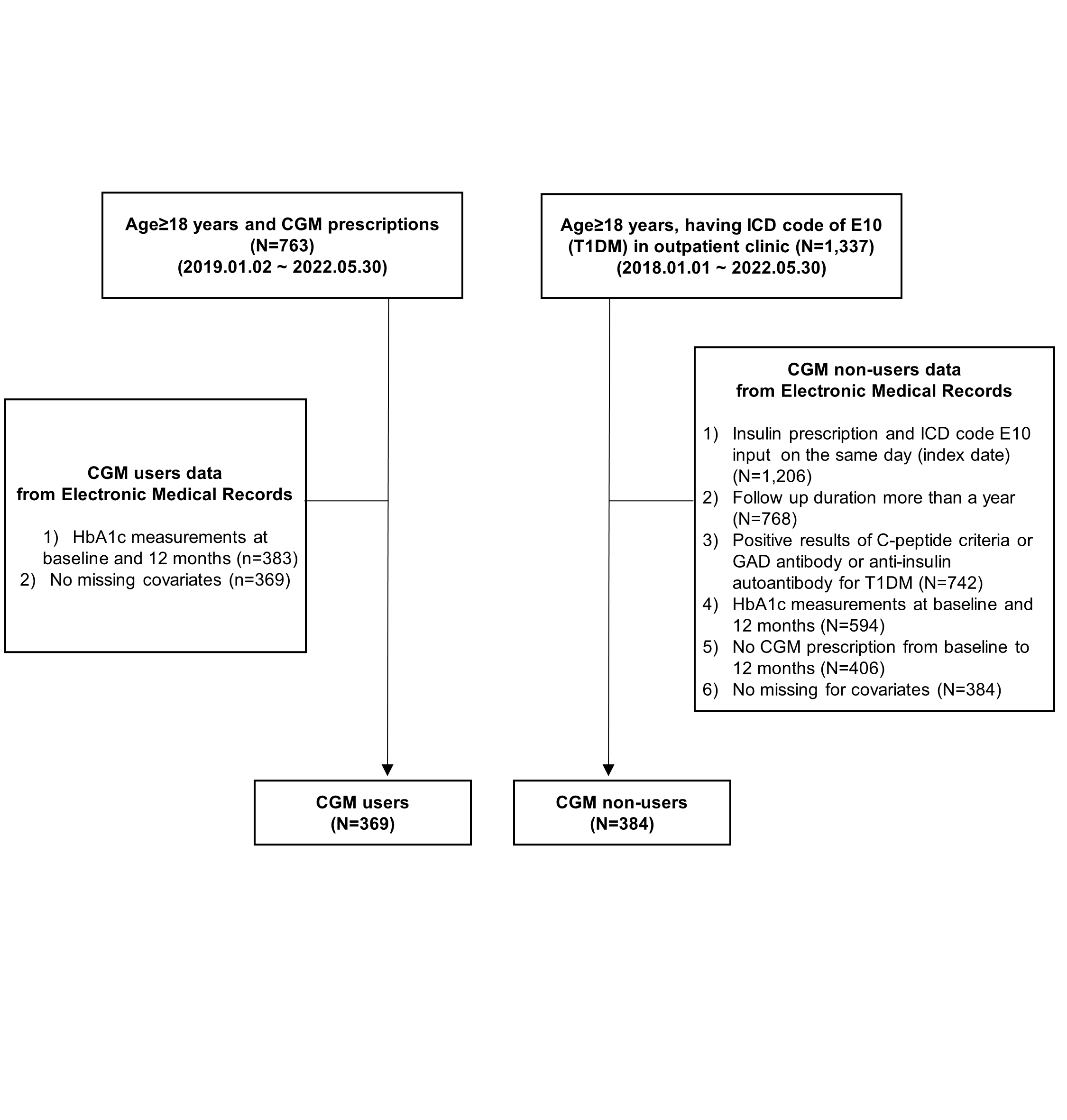

Supplement: Supplementary Figure 1 — Flow chart of sensitivity analysis. CGM, continuous glucose monitoring; GAD, glutamic acid decarboxylase; HbA1c, glycated hemoglobin; ICD, International Classification of Diseases; T1DM, Type 1 diabetes mellitus; T2DM, Type 2 diabetes mellitus. [file Image_1.tif]
