# Supplementary material for: Impact of continuous glucose monitoring on glycemic control and its derived metrics in type 1 diabetes: a longitudinal study
Source: Front Endocrinol (Lausanne). 2023 May 15;14:1165471. doi: 10.3389/fendo.2023.1165471 (PMC10225713; doi:10.3389/fendo.2023.1165471)
Supplement: Supplementary file 2 [file Table_1.docx]

**Supplementary Table 1. Sensitivity Analysis**

|  | **CGM non-users (n=384)**  **(β, 95% CI)** | **CGM users (n=369)**  **(β, 95% CI)** | **P value** |
| --- | --- | --- | --- |
| Overall average reduction of HbA1c (%) in each three months | -0.02 (-0.05, 0.004) | -0.12 (-0.14, -0.09) | <0.001 |
| Average difference of HbA1c (%) in each three months |  |  |  |
| Model 1 | 0 (reference) | -0.09 (-0.13, -0.05) | <0.001 |
| Model 2 | 0 (reference) | -0.09 (-0.13, -0.05) | <0.001 |
| Model 3 | 0 (reference) | -0.09 (-0.13, -0.05) | <0.001 |

*Model 1 is the unadjusted model; Model 2 is adjusted for age, sex, BMI, and baseline HbA1c; Model 3 is further adjusted for eGFR, total cholesterol, HDL, LDL, and TG.

*Abbreviations : BMI=body mass index, CGM=continuous glucose monitoring, CI=confidence interval, eGFR=estimated glomerular filtration rate, HbA1c= hemoglobin A1c, HDL=high-density lipoprotein, LDL=low-density lipoprotein, TG=triglyceride

**Supplementary Table 2. Analysis of baseline characteristics associated with change of HbA1c from baseline to 12 months by multivariable linear regression in CGM user group (N=155)**

| **Variable** | **β (95% CI)** | **P value** |
| --- | --- | --- |
| Age, year | 0.005 (-0.001,0.022) | 0.08 |
| Sex |  | 0.324 |
| Female | Ref |  |
| Male | -0.155 (-0.465, 0.154) |  |
| Baseline HbA1c, % | -0.433 (-0.545, -0.321) | <0.001 |
| BMI, kg/m^2^ | 0.016 (-0.045, 0.076) | 0.611 |
| eGFR, ml/min/1.73 m^2^ | 0.002 (-0.004, 0.008) | 0.547 |
| Total Cholesterol, mg/dL | 0.0002 (-0.001, 0.002) | 0.783 |
| HDL cholesterol, mg/dL | 0.0003 (-0.009, 0.01) | 0.944 |
| LDL cholesterol, mg/dL | 0.002 (-0.003, 0.006) | 0.461 |
| TG, mg/dL | 0.002 (-0.002, 0.005) | 0.414 |
| Duration of diabetes, year | 0.001 (-0.016, 0.018) | 0.89 |
| Total daily dose of insulin, units | 0.003 (-0.005,0.011) | 0.473 |
| Insulin regimen |  | 0.641 |
| Multiple daily injections | Ref |  |
| Continuous subcutaneous insulin infusion | -0.166 (-0.871, 0.538) |  |

*Abbreviations : BMI=body mass index, CGM=continuous glucose monitoring, CI=confidence interval, eGFR=estimated glomerular filtration rate, HDL=high-density lipoprotein, LDL=low-density lipoprotein, TG=triglyceride
